# A comprehensive review of the botany, ethnopharmacology, biochemistry, pharmacology, pharmacokinetics and toxicity of *Filifoliumsibiricum* (L.)Kitam

**DOI:** 10.1186/s13020-021-00471-w

**Published:** 2021-08-23

**Authors:** Shaowa Lv, Qian Qiu, Qiuhong Wang, Haixue Kuang

**Affiliations:** 1 Key Laboratory of Ministry of Education, Department of Pharmacology, Heilongjiang University of Chinese Medicine, No. 24 Heping Road, Harbin, 150040 Heilongjiang Province People’s Republic of China; 2 College of Traditional Chinese Medicine, Guangdong Pharmaceutical University, No. 280 Guangzhou Higher Education Mega Center, Guangzhou, 510006 Guangdong Province People’s Republic of China

**Keywords:** *Filifoliumsibiricum* (L.)Kitam, Ethnopharmacology, Biochemistry, Pharmacology, Pharmacokinetics, Toxicity

## Abstract

*Filifoliumsibiricum* (L.)Kitam (*F.* *sibiricum*), a compositae plant, is especially used to inhibit drug-resistant bacteria in folk medicine. Modern pharmacological studies also confirmed a variety of pharmacological properties about sedative activities, antibacterial activity, anti-inflammatory activity, analgesic activities, antitussive and asthma relieving. In this paper, the research progress of *F.* *sibiricum* in botany, ethnopharmacology, phytochemistry, pharmacology, pharmacokinetics and toxicology was reviewed. Prospects for future investigation and application of this herb were also discussed. Information on *F.* *sibiricum* was gathered from various sources, including books on traditional Chinese herbal medicine and scientific databases such as PubMed, Google Scholar, Science Direct, Baidu Scholar, CNKI and other professional websites. The results indicate that ~ 66 chemical compounds were isolated and identified from *F.* *sibiricum.* Among them, flavonoids are generally considered to be the main bioactive and characteristic ingredients. *F.* *sibiricum* is a traditional Chinese medicine with pharmacological activities such as the immune system, nervous system, respiratory system and cardiovascular and cerebrovascular systems. Most importantly, we should concentrate on developing new drugs related to *F.* *sibiricum*, so as to exert greater potential for treatment.

## Introduction

*Filifoliumsibiricum* (L.)Kitam is known as “Xian Yeju” or “Tu Maohao” in Chinese. It is a necessary herb with other aliases, namely *Tanacetum* *sibiricum* L (Latin) included in the national compilation of Chinese herbal medicine and Siberian Filifolium herb recorded in the dictionary of traditional Chinese medicine [1]. *F.* *sibiricum* recorded in Chinese Materia Medica (Zhong Hua Ben Cao) is regarded by farmers as a herb, but it plays a role in ethnomedicine. It is very important to know if the pharmacological research of *F.* *sibiricum* can be used to verify its traditional uses. Modern pharmacological studies in vivo and in vitro have increasingly confirmed that the traditional use of *F.* *sibiricum* includes sterilization, anti-inflammation, clearing away heat and toxic materials. At the same time, the drug is widely used in Inner Mongolia Medical College, Qiqihar Medical College and Affiliated Hospital of Heilongjiang University of Traditional Chinese Medicine to treat carbuncle, otitis media, chronic bronchitis, angina pectoris, irregular menstruation and other diseases. Drug-resistant bacterial infection is becoming increasingly serious in the clinic, and there is a lack of effective treatment. The over-the-counter and uncontrolled use of antibiotics has led to the rapid development of antibiotic resistance of strains, which has introduced inconvenience to clinical work [2]. Existing studies have shown that *F.* *sibiricum* has inhibitory effects on drug-resistant *Staphylococcus* *aureus*, *Dysentery* *bacillus* and *Escherichia* *coli*. In recent years, with the increasing resistance of bacteria to traditional antibiotics, research on anti-infective drugs has been a prominent and difficult topic in global drug research. Currently, research on anti-infective drugs is facing unprecedented challenges, and the main problem is the growing drug resistance of bacteria. This is not alarmist talk. Therefore, it is of significance to promote research on *F.* *sibiricum* as a substitute for antibiotics. Studies certify that developing some new anti-infective active substances from natural herbs for the new drugs to treat drug-resistant bacterial infections is a direct and effective method. It provides the possibility for improving the situation of antibiotic abuse and drug-resistant superbugs. *F.* *sibiricum*, as effective and seriously underestimated resources, should be valued and exploited.

A complete review is necessary to advance research on *F.* *sibiricum*. Therefore, this paper systematically reviews the research progress in the botany, phytochemistry, pharmacology, pharmacokinetics and toxicity of *F.* *sibiricum*. It is hoped that this review will promote the further study of pharmacological effects and mechanisms related to the therapeutic effects. It can provide basis for further research and development of new drugs [3]. We believe that the present article will provide a better understanding of it and its properties.

## Botany and ethnopharmacology

### Botany

**Fig. 1:**
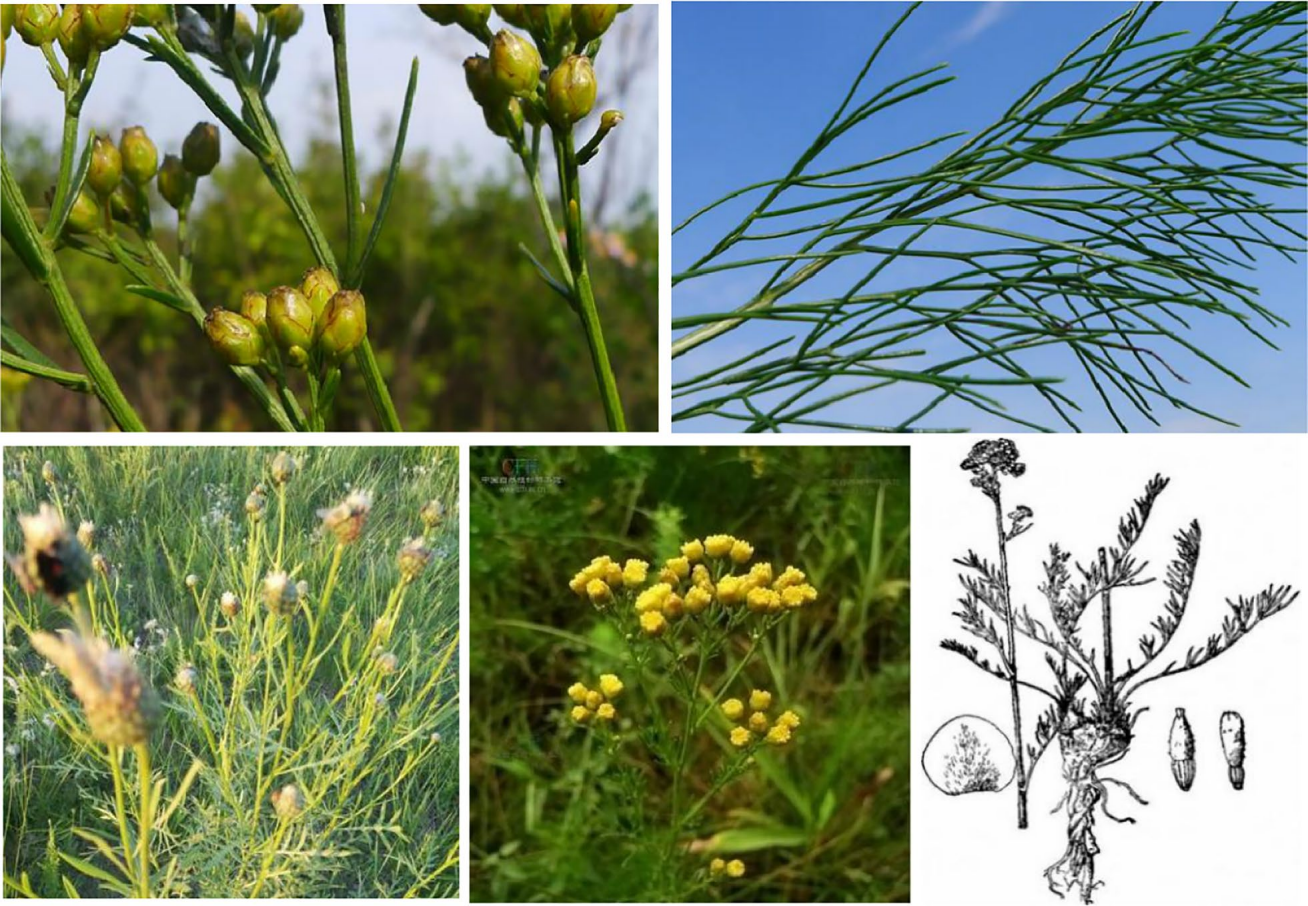
Morphological characteristics of *Filifoliumsibiricum* (*L.*)*Kitam*

This herb grows up to 50 cm in height as a perennial herbaceous plant. The principal botanical characteristics of *F.* *sibiricum* are highlighted here, including the roots, leaves, flowers and fruits. Its roots are sturdy and lignified, up to 20–60 cm in length. The stems are erect, tufted and dense, with bases densely covered with thick fibrous sheaths. This herb is unbranched or has little branching and is also glabrous and striped [4]. The basal leaves have long petioles, and they are obovate or long-circle in shape with a length of 10–20 cm and a width of 5–6 cm. However, cauline leaves are smaller. In addition, each leaf is bipinnate or tripinnate, and leaf blades are striate or filamentous. The head is arranged in a corymb at the top of the stem, and pedicels grow up to 1–11 mm in length. The involucre of the bracts is ovate, brown and herbaceous. The corolla appears tubular with slight narrowing at the top. The achene is obovate or elliptical, black and glabrous with two ventral stripes [5]. The herbs are relatively conspicuous (see Fig. 1). The flowering and fruiting period probably occurs from June to September. The plants begin to germinate in the middle or the second half of May, blooming in July and ripening in early August. Yellow flowers appear throughout the mountains and plains in mid to late September [6]. With frost, the achenes fall, the leaves start to turn red, and the plants begin to wither.

**Fig. 2:**
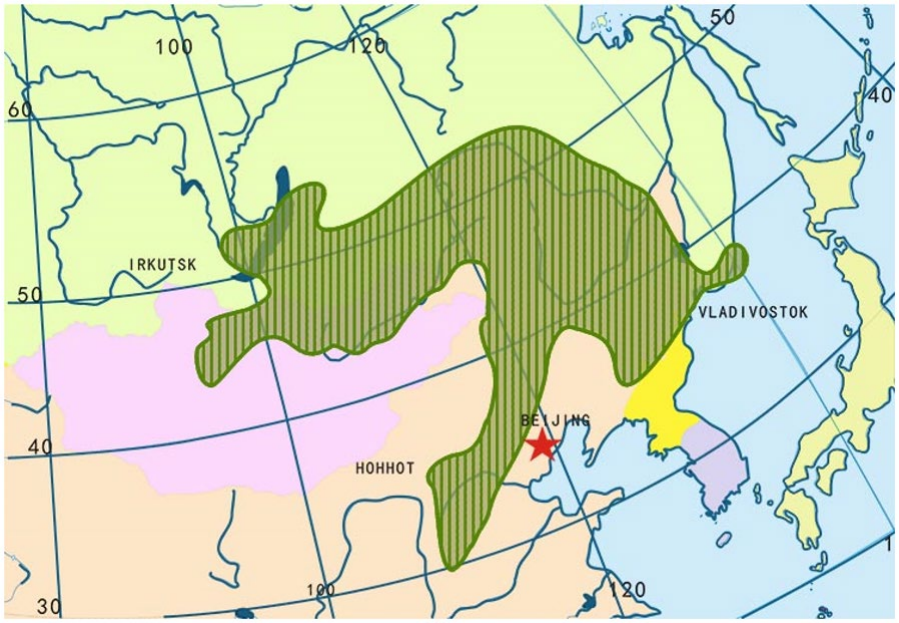
Distribution diagram of *F.* *sibiricum* grassland

*F*. *sibiricum* is a temperate zone hardy medium-early perennial herbaceous plant. This herb prefers chilly and moist places, so it mostly grows near hillsides, meadows and hilly stony lands or in shady wet places under valley groves [7]. *F.* *sibiricum* is a slow-growing and long-lived medium xerophyte and grows mainly based on seed reproduction. With the peculiarity of tolerating the cold, this herb thrives in a cold and humid climate and grows on mountain slopes and in grasslands [8]. This herb is particularly prevalent in Heilong jiang, Jilin, Hebei, Shanxi, Shandong, Xiaxi, Gansu, Inner Mongolia and other provinces of China. It is also distributed in Russia, Korea, Japan and other regions [9]. *F.* *sibiricum* grassland should be distributed in the low hill areas east and west of the Great Khingan Ranger, Hulunbeier, eastern Xilingol Steppe and northern Songnen Plain in China. *F.* *sibiricum* grassland is a widely distributed dominant group in the forest steppe zone, and its distribution range is roughly between 100 and 132 east longitude and 37 and 54 north latitude. The distribution of this species is slightly bowl-shaped (see Fig. 2).

With increasing growth, the terrestrial biomass of *F.* *sibiricum* gradually increased to a maximum on the first and middle of August, exhibiting a logarithmic growth pattern [10]. The above-ground biomass of all plant groups observed in the middle of September decreased, which was mainly caused by the gradual senescence of plants as the temperature dropped and an increasing number of dead leaves dropped [11]. The plants began to transfer nutrients from the above-ground parts to the roots after the middle of August. Thus, the quantity of underground biomass first decreased and then increased [12]. The root biomass provides a basis and guide for the picking, drying and utilization of *F.* *sibiricum.*

### Ethnopharmacology

*F.sibiricum* has been considered as a powerful drug with extensive biological and pharmacological effects for a long time, and it plays an indispensable role in Chinese health care. As documented in the Compendium of Materia Medica, the achievements of herbology in China over the past two thousand years were systematically summarized and reflected the scientific achievements of contemporary Chinese medicine [13]. This herb also has the effect of clearing away heat and toxic materials, regulating menstruation and calming nerves, and is mainly used for treating fever, palpitations, irregular menstruation and carbuncle and ulcers. According to the records in Inner Mongolia Chinese Herbal Medicine, it can be used for clearing away heat and toxic materials and cooling blood. So it can be used for treating high fever and carbuncle of infectious disease and numbness or stinging pain caused by chronic blood diseases. This herbal medicine can be made into a paste for external use with whole grass. Nevertheless, according to the national compendium of Chinese herbal medicine, special attention should be paid to the pain when using this herb for the first time. Therefore, it is recommended to take this herbal medicine with analgesics [14]. As a cheap resource and an easy-to-grow herb, it appears in the prescription of traditional Chinese medicine for thousands of years. At present, there are many pharmaceutical drug products made of the active ingredients of *F.* *sibiricum* in the market, including injections, plasters, suppositories, dropping pills, and capsules [15]. Clinically, pneumonia, children’s upper respiratory tract infections, tracheitis, tonsillitis and so on have been treated by injection. Generally speaking, external plasters is used to treat chronic ulcers and other surgical infections of the lower limbs, and usually cures ulcer within one week after two doses [16]. In a word, the herb is the main medicine for treating infectious diseases, high fever, insomnia and neurasthenia. It also has a significant effect on excessive menstruation or irregular menstruation. External use is extremely effective in treating swollen carbuncle and scabies, such as chronic ulcers of the lower limbs, otitis media and other surgical suppurative infectious diseases [17].

## Phytochemistry

*F.sibiricum*, used as a medicinal herb, contains over 60 chemical constituents. The main chemical constituents have been isolated and roughly identified, including flavonoids, volatile oils, triterpene saponins, coumarins, steroidal glycosides, tannins, organic acids, saccharides, polysaccharides and so on. Among them, flavonoids, coumarins and volatile oils are the most important and abundant bioactive components in this Chinese herbal medicine [18]. According to the available phytochemical investigations, compounds have been isolated from part or whole extracts of *F.sibiricum*, and are considered to be promising components for future evaluation. However, quantitative detection of the active ingredients needs further study.

### Flavones

Recent studies have confirmed that *F.* *sibiricum* is rich in flavonoids, which is considered as the active components of this plant medicine. The biological and biochemical activities of these components have been studied by many experiments. Flavonoids are antioxidants, which can eliminate superoxide radical, prevent cell ageing, enhance immunity and prevent cancer. Flavonoids can also improve blood circulation. It has been found that flavonoids also have many biological and pharmacological effects, such as antipyretic, analgesic, anti-inflammatory, antibacterial, relieving respiratory symptoms, and treating allergy and diabetic complications. Anti-infective active ingredients of *F.* *sibiricum* were isolated by different methods. For example, one flavonol, quercetin (44), was obtained from aerial parts of the plant by using column chromatography with macroporous adsorption resin, column chromatography with silica gel, ODS column chromatography and HPLC and was identified with NMR in 2011.

Up to now, approximately 25 flavones from *F.* *sibiricum* have been isolated and identified [19]. It was found that flavonoids, flavonols and flavonols were the main components. Moreover, various flavonoids, namely, 3,6-dimethoxy-quercetagetin (33), homoorientin (40), isovitexin (41), apigenin-6-arabinosyl-8–glucoside (42), apigenin-3′-methoxy-7-*O*-rutin glycoside (43), tricin (52), 5,7-dihydroxy-chromone (53), capillarisin (58) [20], and six flavonols, namely, quercetin (44), hyperin (45), isoquercitrin (46), isorhamnetin-3-*O*-glucoside (47), isosporin-3-*O*-rutin glycoside (48), quercetin-3-*O*-rutin-7-*O*-glucoside (49), and five dihydroflavones, namely, filifolin (34), eriodictyol (35), 5,7,4′-trihydroxy-6-methoxy-flavanone (36), heriodictyol (51), and eriocitrin (50), have been isolated from *F.* *sibiricum* [21]. The chemical structures of these compounds are presented in Fig. 2. The methods of separation and identification are demonstrated in Table 1.

**Table 1:** Chemical component, isolated and identified methods and pharmacology activity on *Filifoliumsibiricum* (*L.*)*Kitam*

No.	Chemical type	Chemical component	Chemical formula	Isolated and identified methods	Pharmacology activity	References
33	Flavonoid	3,6-dimethoxyquercetagetin	C_17_H_14_O8	Polyamide column chromatography	Anti-bacteria, anti-oxidation	[28]
34		filifolin	C_16_H_14_O_7_	Polyamide column chromatography	Anti-bacteria	[28]
35		Eriodictyol	C_15_H_12_O_6_	Polyamide column chromatography	Anti-oxidation, anti-radiation, blood lipid and blood glucose lowing	[28]
36		5,7,4′-Trihy-droxy-6-methoxy-flavanone		Polyamide column chromatography, silica gel column chromatograph, ODS column chromatography	Anti-depression	[29]
37		4-Hydroxyacetophenone	C_8_H_8_O_2_	Polyamide column chromatography, silica gel column chromatograph, ODS column Chromatography	Cholagogue, anticorrosion	[29]
38		Protoeatechuic-acid-methyl-este	C_8_H_8_O_4_	Polyamide column chromatography, silica gel column chromatograph, ODS column Chromatography	Anti-bacterial, anti-inflammatory, anti-oxidative, neuroprotective	[29]
39		l-2-*O*-methyl-chiro-inositol		Polyamide column chromatography, silica gel column chromatograph, ODS column chromatography	Anti-hyperlipidemic	[29]
40		Homoorientin	C_21_H_20_O_11_	Macroporous adsorption resin column chromatography, silica gel column chromatography, ODS column chromatography and high performance liquid chromatography	Anti-oxidation, anti-inflammation, anti-virus, anti-tumor, analgesia, neuroprotection and heart protection	[30]
41		Isovitexin	C_21_H_20_O_10_	Macroporous adsorption resin column chromatography, silica gel column chromatography, ODS column chromatography and high performance liquid chromatography	Anti-bacterial, anti-viral, anti-inflammatory, liver protection	[30]
42		Apigenin-6-arabinosaccharide -8-glucoside	C_26_H_28_O_14_	Macroporous adsorption resin column chromatography, silica gel column chromatography, ODS column chromatography and high performance liquid chromatography	Anti-inflammation	[30]
43		Apigenin-3′-methoxy-7-*O*-rutin glycoside	C_28_H_32_O_15_	Macroporous adsorption resin column chromatography, silica gel column chromatography, ODS column chromatography and high performance liquid chromatography	Anti-inflammation	[30]
44		Quercetin	C_15_H_10_O_7_	Macroporous adsorption resin column chromatography, silica gel column chromatography, ODS column chromatography and high performance liquid chromatography	Expectorant, anti-tussive, anti-inflammation. Swelling diuresis reduction, clear heat and detoxify, immunity increasing. Blood pressure and blood lipid lowering, coronary arteriosclerosis, coronary heart disease, angina pectoris, myocardial ischemia, arrhythmia prevention	[30]
45		Hyperoside	C_21_H_20_O_12_	Macroporous adsorption resin column chromatography, silica gel column chromatography, ODS column chromatography and high performance liquid chromatography	Anti-tumor, anti-oxidation, anti-depression, heart or brain ischemia protection, liver protection, immune regulation	[30]
46		Isoquercitrin	C_21_H_20_O_12_	Macroporous adsorption resin column chromatography, silica gel column chromatography, ODS column chromatography and high performance liquid chromatography	Anti-hypertension, anti-depression, sedation, anti-thrombosis, anti-oxidation, anti-aging	[30]
47		Isorhamnetin-3-*O*-glucoside	C_22_H_22_O_12_	Macroporous adsorption resin column chromatography, silica gel column chromatography, ODS column chromatography and high performance liquid chromatography	Anti-oxidation, anti-apoptosis, neuroprotection	[30]
48		Isorhamnetin-3-*O*-rutinoside	C_28_H_32_O_16_	Macroporous adsorption resin column chromatography, silica gel column chromatography, ODS column chromatography and high performance liquid chromatography	Anti-oxidation, anti-tumor, anti-inflammatory, anti-viral, anti-allergy, cardioprotection	[30]
49		Quercetin -3-*O*-rutin sugar -7-*O*-glucoside	C_33_H_38_O_25_	Macroporous adsorption resin column chromatography, silica gel column chromatography, ODS column chromatography and high performance liquid chromatography	Anti-inflammation,immunity increasing, heart protection	[30]
50		Eriocitrin	C_27_H_32_O_15_	Macroporous adsorption resin column chromatography, silica gel column chromatography, ODS column chromatography and high performance liquid chromatography	Anti-oxidation, blood lipid lowering	[30]
51		Eriodictyol	C_15_H_12_O_6_	Macroporous adsorption resin column chromatography, silica gel column chromatography, ODS column chromatography and high performance liquid chromatography	Anti-oxidant	[30]
52		Tricin	C_17_H_14_O_7_	Extraction, silica gel column chromatography, polyamide column chromatography, recrystallization	Colon cancer prevention, anti-oxidation	[35]
53		5,7-Dihydroxychromone	C_9_H_6_O_4_	Extraction, silica gel column chromatography, polyamide column chromatography, recrystallization	Neuroprotection	[35]
54		Capillarisin	C_16_H_12_O_7_	Extraction, silica gel column chromatography, polyamide column chromatography, recrystallization	Anti-oxidant, anti-inflammatory and anti-tumor	[35]
55		*P*-acetyl-phenol-beta-d-glucoside		EtOH extract, macroporous adsorption resin column chromatography, silica gel column chromatography, ODS column chromatography HPLC		[36]
56		4-(1-Hydroxyethyl)-phenol-1-*O*-β-d-glucoside		EtOH extract, macroporous adsorption resin column chromatography, silica gel column chromatography, ODS column chromatography HPLC		[36]
57	Triterpenoid saponin/Steroidal glycosides	Daucosterol	C_35_H_60_O_6_	Polyamide column chromatography, silica gel column chromatograph, ODS column chromatography	Anti-tumor, anti-oxidation and nerve protection	[35]
58		β-Sitosterol	C_29_H_50_O	Extraction, silica gel column chromatography, polyamide column chromatography, recrystallization	Blood lipid lowering, anti-inflammatory,anti-bacterial, anti-oxidant, anti-tumor, antipyretic and analgesic, gastric mucosa protection,balance regulation of bone metabolism	[35]
59	Coumarins	Scopolin	C_16_H_18_O_9_	EtOH extract, macroporous adsorption resin column chromatography, silica gel column chromatography, ODS column chromatography HPLC	/	[36]
60		Scopoletin	C_10_H_8_O_4_	Extraction, silica gel column chromatography, polyamide column chromatography, recrystallization	Blood pressure lowering, blood fat reducing, anti-inflammation, anti-tumor, antipyretic	[36]
61	Alkaloids	Adenosine	C_10_H_13_N_5_O_4_	EtOH extract, macroporous adsorption resin column chromatography, silica gel column chromatography, ODS column chromatography HPLC	Vasodilation, treatment of heart disease	[36]
62	Aromatic species	Dibutyl phthalate	C_16_H_22_O_4_	EtOH extract, macroporous adsorption resin column chromatography, silica gel column chromatography, ODS column chromatography HPLC		[36]
63		Diisobutyl phthalate	C_16_H_22_O_4_	EtOH extract, macroporous adsorption resin column chromatography, silica gel column chromatography, ODS column chromatography HPLC		[36]
64		Phloroglucinol	C_6_H_6_O_3_	Polyamide column chromatography, silica gel column chromatograph, ODS column chromatography	Smooth muscle relaxation, antispasticity, progesterone regulation, fetal protection	[29]
65	Diphenylpyrones	Mangiferin	C_19_H_18_O_11_	EtOH extract, macroporous adsorption resin column chromatography, silica gel column chromatography, ODS column chromatography, UV spectrum and IR spectrum.C	Anti-tumor, anti-leukemia, analgesia, antidiabetic, anti-inflammatory, cough relief, immune regulation, inhibition of lipid peroxidation	[36]

### Volatile oils

In an experiment, the extract of *F.* *sibiricum* extracted by water distillation was analyzed by gas chromatography–mass spectrometry (GC–MS). 25 volatile oils were identified from extracts of *F.* *sibiricum*, including 12 monoterpenes, 7 sesquiterpenes and 6 other compounds. The identified components accounted for 55.6% of the total volatile oil in this plant [22]. Phytochemical investigations of *F.* *sibiricum* have shown the presence of volatile oils such as β-ocimene (1), dihydrocarveol (2), P-cymene (3), 1,5-hexadiene (4), perillaldehyde (5), aryophyllene (6), trimethylcyclopentadiene (7), p-hydroxyacetophenone (8), furaldehyde (9), linalool (10), α-elemene (11), α-farnesene (12), β-elemene (13), r-terpinene (14), *cis*-caryophyllene (15), *trans*-farnesene (16), verbenone (17), 2-P-immunol (18), benzyl alcohol (19), methylisoeugenol (20), eugenol (21), isoeugenol (22), and 4-methyl-benzaldehyde (23). In addition, chemical constituents such as perillaldehyde, β-pinene and eugenol were isolated from extracts, and their antibacterial activities were further studied. Moreover, some volatile oils also were found, including ocimene (24), β-pinene (25), limonene (26), myrtenal (27), isobornyl acetate (28), geranyl acetate (29), 4-butoxy-3-methyl-2-butanone (30), α-curcumene (31), and α-trans-β-bergamotene (33). In summary, a total of 32 volatile oils (1–32) had been isolated from extracts of *F.* *sibiricum.* The volatile oils from *F.* *sibiricum* were displayed in Table 2 [23].

**Table 2:** Chemical constituents of volatile oil of *Filifoliumsibiricum* (L.)Kitam

No	Chemical component	molecular weight	Chemical formula	Pharmacology activity and application	References
1	β-Ocimene	136.24	C_10_H_16_	Essence	[22]
2	Dihydroearveol	154.08	C_10_H_20_O	/	[22]
3	P-Cymene	134.22	C_10_H_14_	Solvent, synthetic resin, metal liniment	[22]
4	1,5-Hexadiene	82.14	C_6_H_10_	Standard of chromatographic analysis, chemical materials	[22]
5	Perillaldehyde	150.22	C_10_H_14_O	Anti-cancer, bacteriostat, insecticide, anti-allergies, anti-inflammation, essence	[22]
6	β-Caryophyllene	204.36	C_15_H_24_	Local anaesthesia, colitis treatment, essence, spice	[22]
7	Trimethyl cyclopenadiene	108.18	C_8_H_12_	Chemical materials	[22]
8	Phydroxyacetophenone	136.14	C_8_H_8_O_2_	Cholagogue, antiseptic substance, organic material	[22]
9	2-Furaldehyde	96.09	C_5_H_4_O_2_	Organic material	[22]
10	Linalool	154.25	C_10_H_18_O	Antiviral, antibacterial, sedative,anti-odor, anti-caries, insecticide	[22]
11	α-Elemene	204.35	C_15_H_24_	Anticancer, apoptosis-inducing, cells damage	[22]
12	α-Farnesene	204.35	C_15_H_24_	Spice	[22]
13	β-Elemene	204.35	C_15_H_24_	Anticancer, apoptosis-inducing, cells damage	[22]
14	r-Terpinene	136.23	C_10_H_16_	Spice	[22]
15	*Cis*-earyophylene	204.35	C_15_H_24_	Antisepsis, insecticide	[22]
16	*Trans*-farnesene	204.35	C_15_H_24_	Spice	[22]
17	Verbenone	150.22	C_10_H_16_O	Liver protection, antisepsis, detoxication, calm, skin care	[22]
18	2-*P*-eymenol	150.07			[22]
19	Benzyl alcohol	108.14	C_7_H_8_O	Solvent, preservative, photographic developer, essence	[22]
20	Methyl eugenol	178.23	C_11_H_14_O_2_	Spice	[22]
21	Ougenol	164.20	C_10_H_12_O_2_	Antisepsis, spice, spice	[22]
22	Isoougenol	164.20	C_10_H_12_O_2_	Antisepsis, spice, spice	[22]
23	4-Methylbenzaldehyde	120.15	C_8_H_8_O	Organic material	[22]
24	Ocimene	136.24	C_10_H_16_	Spice	[23]
25	β-Pinence(beta-pinene)	136.23	C_10_H_16_	Spice	[23]
26	Limonene	136.23	C_10_H_16_	Relieving cough, eliminating phlegm, antisepsis, cholagogue, promoting digestion function, solvent	[23]
27	Myrtenal	150.22	C_10_H_14_O	Spice, essential oil	[23]
28	Isbornyl aeetate	196.19	C_12_H_20_O_2_	Organic material	[23]
29	Geranyl aeetate	196.23		/	[23]
30	α-Eurcumene	202.16		/	[23]
31	4-Butoxy-3-methyl-2-butanone	158.39		/	[23]
32	α-*Trans*-β-bergamotene	204.18	C_12_H_8_O_4_	Organic material	[23]

### Coumarins

Besides the volatile oils, flavonoids and coumarin mentioned above were also an important component. Scopolamine (60) and Scopolamine (59) had been isolated from *F.* *sibiricum*, belonging to coumarin. Recently, scopoletin (60) was listed as one of the most significant coumarins due to its anti-inflammatory, analgesic, diuretic and antiasthmatic properties. Therefore, the extraction of scopolamine had positive significance. There were some quantitative studies on the components of total flavonoids in *F.* *sibiricum* by ultraviolet spectroscopy and colorimetry [24]. However, there were few quantitative research reports of other components in *F.* *sibiricum* [25]. The methods and components of separation and identification were demonstrated in Table 1.

### Others

**Fig. 3:**
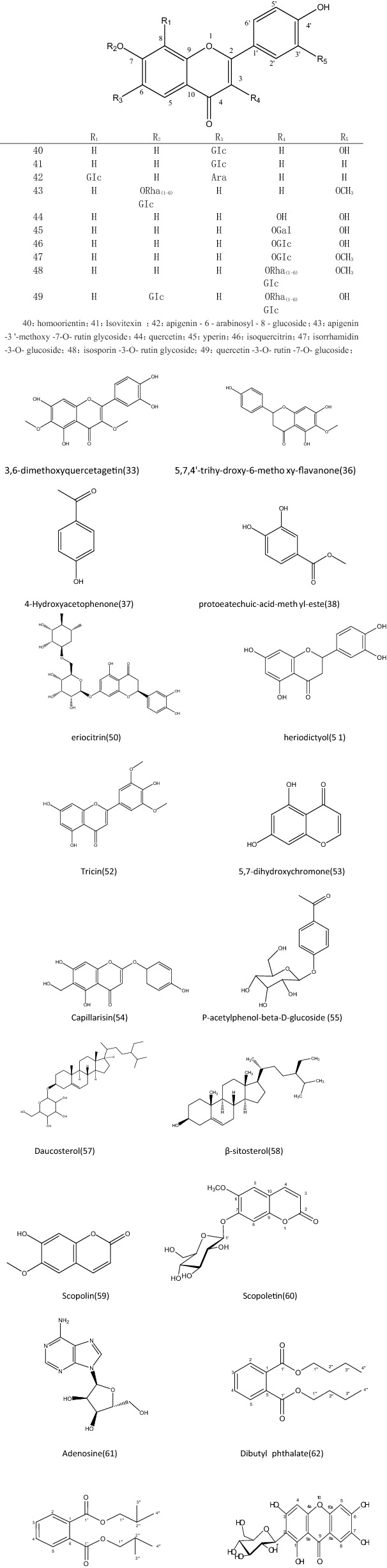
The chemical structures of chemistry compounds of *Filifoliumsibiricum* *(L.)Kitam*

A total of five other compounds had been isolated from *F.* *sibiricum.* Aromatic compounds including ortho-phthalate (62), diisobutyl-phthalate (63), and phloroglucinol (64) had been isolated from *F.* *sibiricum*. Alkaloids compounds namely, adenosine (61), triterpenoid saponins and steroidal glycosides compounds namely, daucosterol (57) [26], β-sitosterol (58), and diphenylpyrones compounds namely, mangiferin (65), had also been found [27]. The separation and identification methods and the pharmacological activity were demonstrated in Table 1. The chemical structures of these constituents were shown in Fig. 3

## Pharmacological activities

In the past decades, the pharmacological effects of the *F.* *sibiricum* based on traditional uses have been extensively studied in ethnic medicine. According to records in “Inner Mongolia herbal medicine”, it was used to treat high fever caused by infectious diseases, carbuncles, blood pain, irregular menstruation, otitis media and others. Modern pharmacological studies have found that this plant has a good effect on improving otitis media and bronchitis. Furthermore, these effects are closely related to the anti-inflammatory properties of this herb. Pharmacological studies have demonstrated that many of the compounds in *F.* *sibiricum* showed good bio-activities in vivo or in vitro. Various studies have confirmed that this herb had antibacterial and bactericidal effects. Moreover, antipyretic analgesic effects as well as effects for dissolving phlegm and arresting cough have been investigated in animal models. The different chemical constituents from *F.* *sibiricum* will lay a good foundation for further drug development.

### Antimicrobial activity

The World Health Organization has warned that the rise in bacterial drug resistance has reached a level of crisis. Antibiotic resistance is a serious emergent global issue. Strengthening the research on antimicrobial resistance of *F.* *sibiricum* will solve this problem and bring good news to the whole world. The advantage of *F.* *sibiricum* is in treating drug-resistant bacteria. In the traditional medical treatment in China, *F.* *sibiricum* was used as a heat-clearing and detoxifying drug. Lingyan Lv et al. [28] found that flavonoids with an anti-inflammatory effect are the main components of the antibacterial action of *F.* *sibiricum*. Therefore, ShaoWa Lv et. al showed that flavonoids such as rhamnetin 3-*O*-rutin, isoorientin, isovitexin, myricetin, scopoletin, isorhamnetin 3-*O*-β-d-glucoside and quercetin-3-rutin-7-murine *O*-glucoside were the main material basis for the bacteriostatic effect of *F.* *sibiricum* by grey correlation analysis. Many investigations indicated that the growth and reproduction of clinically common human pathogenic bacteria including *Staphylococcus* *albicans*, *Pseudomonas* *aeruginosa*, *Escherichia* *coli*, beta-haemolytic Streptococcus, Pneumobacillus, *Proteusbacillus* *vulgaris*, *Trichophyton* *rubrum,* *Shigella* *flexneri,* *Salmonella* *paratyphi* *a,* *Staphylococcus* *aureus* and *Shigella* *dysenteriae* can be suppressed by the ethanol extraction of *F.* *sibiricum* in the vitro antibacterial tests. Qiuhong Wang et al. [29] investigated inhibitory effect of *F.* *sibiricum* for drug-resistant bacteria, using Ceftriaxone sodium as positive control in vitro. The studies indicated that the extraction (0.5 g/L) of *F.* *sibiricum* with 50% ethanol had an obvious bactericidal effect on drug-resistant Staphylococcus aureus. This bactericidal effect in *F.* *sibiricum* group was better than in the positive control group. Yujie Liu et al. studied that *F.* *sibiricum* (576, 288, 144 mg/kg) had bacteriostatic and bactericidal effects on 12 common pathogenic bacteria. The results proved that its bacteriostatic effect was better than Shuanghuanglian oral liquid group. Moreover, it still had bacteriostatic and bactericidal effects on drug-resistant Pseudomonas aeruginosa, but the cephalosporin positive control grouphad no effect on it. It also has bacteriostatic and bactericidal effect on drug-resistant *Escherichia* *coli* and drug-resistant *Klebsiella* *pneumoniae.*

Currently, research on anti-infective drugs is facing unprecedented challenges, and the main problem is that bacterial infection after operation leads to fever and even death. *F.* *sibiricum* has a protective effect on surgical infection of drug-resistant *Staphylococcus* *aureus* and reduces mortality. In vivo antibacterial experiment, mice in the bacterial infection group were given 0.5 mL penicillin-resistant *Staphylococcus* *aureus* solution (1 × 109/mL), while the mice in the *F.* *sibiricum* group were given intragastric administration of total flavonoids (442 mg/kg). The number of deaths in mice within 72 h after infection was observed and recorded. The results showed that all the mice in the bacterial infection group died within 24 h after infection with penicillin-resistant *Staphylococcus* *aureus*. The death protection rate of total flavonoids of *F.* *sibiricum* was 60% (P < 0.01). It is suggested that the total flavonoids of *F.* *sibiricum* can significantly inhibit the death of mice caused by penicillin-resistant *Staphylococcus* *aure*. Therefore, it is of great clinical significance.

**Fig. 4:**
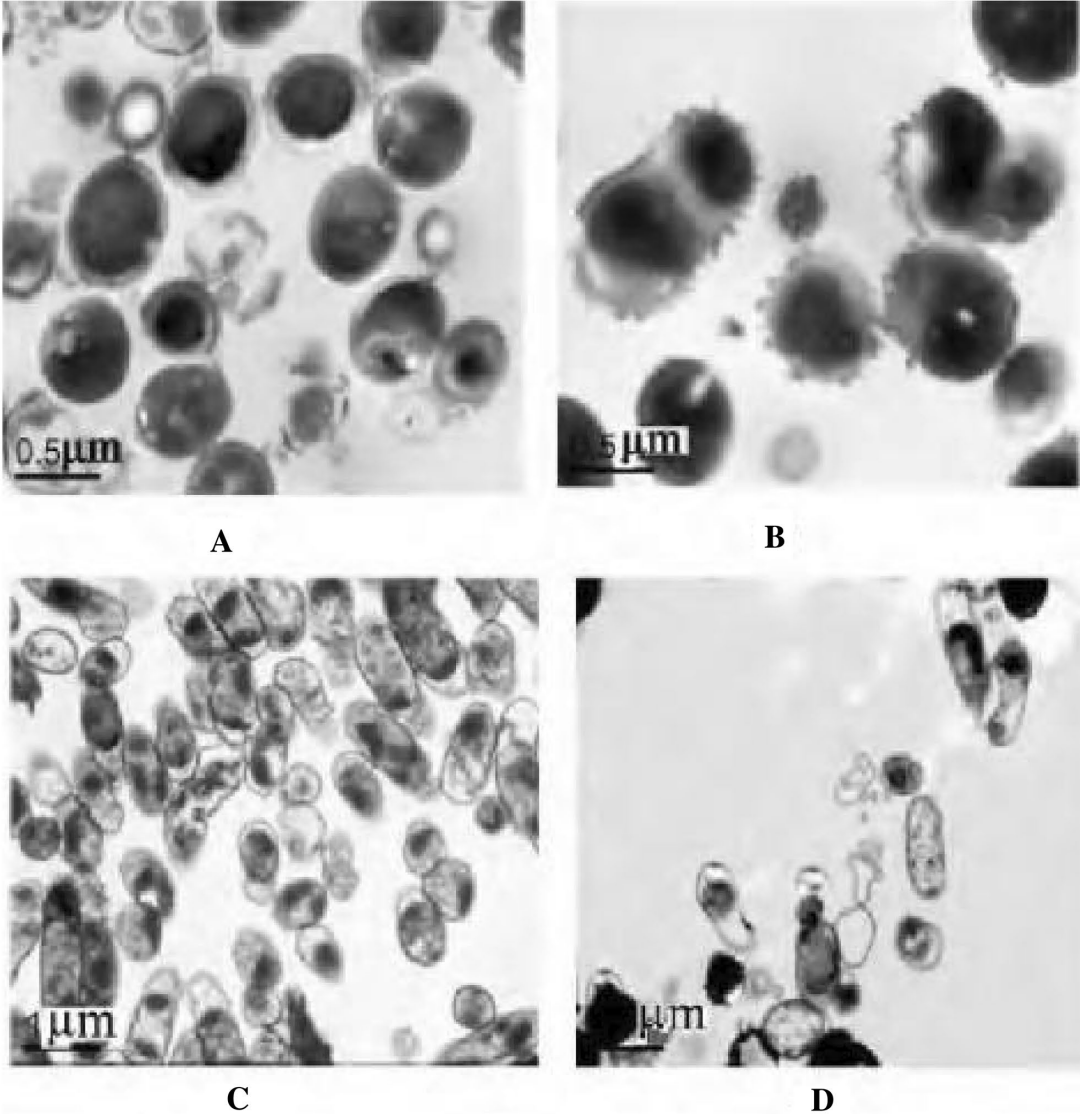
Electron microscope images of the effective anti-infection sites of *Filifoliumsibiricum* *(L.)Kitam* afterinteraction with *Staphylococcus aureus* (× 13,000) and *Escherichia* *coli* (× 11,500). **A** *Staphylococcus aureus*. **B** *Staphylococcus aureus* after the action of *Filifoliumsibiricum* (L.)Kitam. **C** *Escherichia coili*. **D** *Escherichia coli* after the action of *Filifoliumsibiricum*(L.) Kitam

Some researchers believe that the antimicrobial mechanisms of *F.* *sibiricum* are generally due to cell membrane or wall damage as well as nucleoli disappearance causing increased permeability of cell membranes or lysis of cell walls, loss of cellular constituents, impairment of structural components and changes in bacterial cell morphology and then reduction in bacterial density [30]. Furthermore, other researchers found that the antibacterial activity of *F.* *sibiricum* against strains was possibly closely related to inhibiting the expression of soluble proteins and the synthesis of nucleic acid. To further explore the antibacterial mechanism of *F.* *sibiricum*, the morphology of *Staphylococcus* *aureus* and *Escherichia* *coli* before and after drug delivery was observed by electron microscopy. As shown in Fig. 4. It was found that the cell wall of the bacteria was severely damaged after interaction with *F.* *sibiricum*, *and* the cytoplasm of the bacteria also showed different degrees of destruction. There are few researches on its antibacterial mechanism, so it should be further studied from molecular point.

As a natural antimicrobial agent, all parts of the *F.* *sibiricum* have few side effects and inhibit or kill bacteria through multiple targets and pathways. It is not easy to produce a drug effective against resistant strains that also has high safety. On the basis of guaranteeing safety and efficacy, attention should be paid to the development and utilization of natural antimicrobial agents from Chinese herbal medicines such as *F.* *sibiricum*. Further developing effective antimicrobial agents and applying antimicrobial agents in a broader field will be the most important problems in the development of antimicrobial materials with a broad spectrum in China in the future.

## Anti-inflammatory activity

The effective part of *F.* *sibiricum* fought inflammation and boosts immunity. A pyelonephritis model was established by unilateral ureteral obstruction and the injection of *Escherichia* *coli* into the bladder. The rats were divided into a model normal group, three dose groups of the anti-infective part of *F.* *sibiricum* and a positive control group. Afterwards, urine *N*-acetyl-amino group glucosaminidase (NAG)/urinary creatinine (UCr) was monitored before administration, and the visceral index and serum interleukin-2 (IL-2) content in the thymus and spleen of rats were monitored after administration. ELISA was used to examine the content of circulating immune complexes to measure the activity of interleukin-2 (IL-2). In addition, renal biopsy was performed to study pathological changes. HaiXue Kang et al. showed that there was no difference in Scr and BUN between the *F.* *sibiricum* groups and the model group. Compared with that of the model group, the left kidney coefficient increased in the high-dose group of *F.* *sibiricum* [31]. The activity of IL-2 was significantly enhanced in the *F.* *sibiricum* groups (P < 0.05). Furthermore, the optical microscopy results showed that the anti-infective part of *F.* *sibiricum* could improve kidney pathological changes. Moreover, *F.* *sibiricum* could reduce glomerular inflammation and improve renal function and had some therapeutic effects on pyelonephritis. At the same time, the herb can improve the immune ability and enhance the function of the immune system [32]. They also divided the experiment into a normal group, a model group and a total flavonoid group by using the mouse ear swelling method and the cotton ball granuloma test for observation. Furthermore, the study showed that the effective anti-infection part of *F.* *sibiricum* had an anti-inflammatory effect.

## Antipyretic and analgesic effect

*F.sibiricum* has the effect of heat clearing and toxicity removal with treatment of the hyperpyrexia of infectious disease. Shaowa lv at el. [33] performed a series of experimental studies to verify whether the anti-infective effect of *F.* *sibiricum* is related to its antipyretic and analgesic effects. Moreover, oral treatment with the ethanol extract of *F.* *sibiricum*, namely, the anti-infective effective part of *F.* *sibiricum* (576, 288 and 144 mg/kg), significantly reduced paw licking in rats experiencing induced pain. Synchronously, in hot plate tests of mice, the high-dose group suggested a significant increase in response latency at 30 min and a most remarkable increase in latency at 60 min compared with the control group following treatment. Therefore, the pharmacological action of *F.* *sibiricum* against infection was related to the extension of the pain domain value of mice and its analgesic effect. However, the exact mechanism of the analgesic activity of this herb needs to be elucidated. In addition to its analgesic effect, *F.* *sibiricum* also showed possible antipyretic effects. The positive control group received aspirin at a daily dose of 0.18 g·kg^−1^, the blank group received water at the same volume at a daily dose of 1.5 ml·kg^−1^, and the medication administration groups received the ethanol extract of *F.* *sibiricum* at a daily dose of 0.403 g·kg^−1^, 0.201 g·kg^−1^, and 0.100 g kg^−1^. After one hour of intragastric administration, rats were injected with 2,4-dinitrophenol subcutaneously on the back, and their temperature was tested every 60 min 4 times. However, the active parts of the *F.* *sibiricum* ethanol extract remarkably did not diminish the body temperature in fevered experimental rats. Nevertheless, in a dry yeast-induced fever rat model, flavonoid dropping pills of *F.* *sibiricum* produced by Chifeng Tianqi Pharmaceutical Co. LTD, with a 15% total flavonoid content, reduced the rat body temperatures to normal levels. At present, because of the few studies and unclear research on the pharmacological action and mechanism of action of this herb, the conclusion is uncertain, and more in-depth research is needed.

### Effects on the respiratory system

*F.sibiricum* is more like drinking as tea used in folk medicine to treat chronic bronchitis. Chronic bronchitis, belonging to “cough” and “phlegm drink” in the categories of traditional Chinese medicine, is a chronic process characterized by coughing, phlegm production, asthma and recurrent attacks in the clinic. In this study, based on traditional clinical experience in the use of *F.* *sibiricum*, the total flavonoids of *F.* *sibiricum* were determined to be the main effective anti-infection compounds through basic study of pharmacodynamic substances. In addition, the antitussive and expectorant pharmacological effects of the selected total flavones were studied, and the pharmacodynamics of the experimental animal models of chronic bronchitis were observed. Experiments with an ammonium hydroxide-induced cough model in mice evaluated the cough-relieving activity of flavone dripping pills (16 g/(kg d), 8 g/(kg d), 4 g/(kg d)) from *F.* *sibiricum* with tablets as a positive drug, and the negative control group was the blank dripping pill solution dissolved in 0.5% sodium carboxymethyl cellulose solution (10 g/kg). Compared with the low-dose group, the high-dose group and positive group remarkably inhibited cough, and the duration of cough induced by chemical stimulation in mice, and the effect in the low-dose, middle-dose and high-dose groups was higher than that of the negative control group. Hence, the results show that flavone dripping pills have obvious antitussive effects. According to the requirements of the new drug registration administration method, the experiment observed the antitussive effect of flavone pills from FS on mice by means of concentrated ammonia spray to provide basic data for clinical research [34]. Moreover, Wang et al. investigated healthy male and female 8–10 weeks old rats that were induced with ammonia to produce a cough model in vivo and administered an oral medication with *F.* *sibiricum* (high-, medium- and low-dose groups with doses of 8 g/kg, 4 g/kg, 2 g/kg, respectively) for 4 days. Finally, the cough incubation period and cough frequency within 3 min were recorded as parameters. The experimental results showed that the cough incubation period of the drug group was significantly different than that of the model group (P < 0.05). Furthermore, it was found that the incubation period of cough in rats was significantly prolonged, and the number of coughs per unit time was low. However, at present, the mechanism of action of the antitussive effect is not clear and needs further study.

In a vivo study of a rat model of chronic bronchitis induced by SO_2_, oral administration of an ethanol extract of *F.* *sibiricum* reduced calcitonin levels (CGRP) and increased IL-2 levels in serum, which alleviated calcification of lung cells or even reduced damage to bronchus and lung functions. In summary, it has been observed that *F.* *sibiricum* can improve the pathological structure of bronchi and lung, such as reducing inflammatory cells and slowing the proliferation of connective tissue. Furthermore, this herb can improve immune ability, enhance immune system function and protect major immune organs to varying degrees, illustrating that its action pathway is related to the regulation and control of immune function in the body. Therefore, it is speculated that through the overall effect on the body, *F.* *sibiricum* can achieve a chronic curative effect of bronchitis to provide a scientific basis for the development of a new drug to treat chronic bronchitis [35].

### Effects on the cardiovascular system

*F.sibiricum* can suppress heart contraction and lower blood pressure. In experiments on the contractile heart function of isolated toads, it was found that a water decoction with an *F.* *sibiricum* concentration of 2.5% reduced the contractile heart function of isolated toads. Nevertheless, the inhibitor can be enhanced by 2 g/mL acetylcholine and countered by 10 g/mL epinephrine [36]. The experimental results showed that a 0.2–0.4 mL injection of *F.* *sibiricum* also inhibited cardiac contraction in the toads*.* After anaesthesia, rabbits repeatedly received *F.* *sibiricum* injections into their ear veins. It was found that their blood pressure decreased and the heart rate inordinately slowed even when there was temporary respiratory centre excitement. However, it is speculated that there would be no obvious change with an oral dose of 20 g for adults. Notably, acute poisoning can lead to respiratory centre failure and damage to the cardiovascular system.

### Other effects

*F.sibiricum* had a sedative effect. In an experiment on sedated hypnosis induced by an extract of *F.* *sibiricum* [37], rats obviously showed a state of closing eyes, lying down and decreasing voluntary activities after intraperitoneal administration. In addition, this herb also has an inhibitory effect on tumours and enhanced effect of immune function.

## Pharmacokinetic study

The study of pharmacokinetics is a way to understand the behavior and mechanism of action in *F.* *sibiricum*. Pharmacokinetic studies can be used to explain and predict valuable phenomena related to drug efficacy and toxicity. A certain amount of the effective part of shade-dried *F.* *sibiricum* was heated and recycled with 50% ethanol to obtain the extract (1.0 g/kg). The extract was given to rats once by gavage. At 5, 10, 15, 30, 60, 90, 120, 150, 180, 240, 360 min after oral administration, blood samples were taken from the fundus artery plexus. Lv et al. [38] used ultra high performance liquid chromatography-mass spectrometry (UHPLC–MS/MS) to determine the content of target components in rat plasma quickly, sensitively, selectively and accurately. Simultaneously, isoorientin, isorhamnetin-3-*O*-β-d-glucoside and scopolin in rat plasma at different times were determined to research pharmacokinetics. The trend of absorption and metabolism of drugs in the body can be seen directly. The purpose of selecting three indexes is to provide data support for the further development of *F.* *sibiricum* in vivo dynamic process and its dosage forms. This study provides a reference for guiding the rational use of drugs in clinic. However, pharmacokinetic studies of the above three compounds are not comprehensive, and further studies are needed. In-depth study can be carried out from the aspects of metabolism and excretion to explore whether these three components and other compounds have undergone chemical structural changes in the body.

## Toxicology

There are few and inadequate studies on the toxicity of *F.* *sibiricum* in the current literature. The acute toxicities, subacute toxicities and LD50 determination of *F.* *sibiricum* have been evaluated. The median lethal dose (LD50) of oral decoction of the *F.* *sibiricum* herb is 520 ml/kg in mice. In a subacute toxicity study, rabbits were given intragastric administration of *F.* *sibiricum* at a dose of 2 ml/kg for 2 weeks. It has been found that the red blood cell and white blood cell counts and the indicators NFN and GPT did not exhibit abnormal changes in heart blood [39]. There were also no abnormal changes in ocular observation and microscopic examination of viscera. The mice were injected with *F.* *sibiricum* several times within 24 h, and the total flavonoids in their body reached 10.6 g/kg. No deaths were observed in the mice. However, additional toxicology studies and large clinical trials are necessary to assess the toxicity of *F.* *sibiricum* Further investigations regarding the long-term toxicity of *F.* *sibiricum* are appreciably necessary. The toxic effects of this herb on patients with underlying conditions or in special populations, such as elderly individuals, children, pregnant women and nursing mothers, have not been determined and should be further assessed.

## Others uses

Dried *F.* *sibiricum* is not only medicinal but also edible. The production of *F.* *sibiricum* grassland for animal husbandry in North China plays an important role in the cultivation of this plant. Research on this kind of grassland has attracted increasing attention in animal husbandry production [40]. Grazing is an important measure of grassland management. Proper grazing can increase the biodiversity of the grassland community, improve the productivity of the grassland, and promote the stable and sustainable development of the grassland [41]. However, overgrazing not only leads to the retrograde succession of grassland communities but also to the degradation of the species, structure and function of grassland vegetation [42]. Therefore, grazing changes the community and population characteristics of grassland vegetation, resulting in different degrees of grassland degradation [43]. In the process of grazing, with increasing grazing intensity through the feeding, trampling and excretion activities of livestock, the species composition, plant community structure, species diversity and vegetation yield of grassland will decrease [44]. At the same time, grassland vegetation will experience retrograde succession, and the physical and chemical characteristics of grassland soil will change accordingly. These changes are only quantitative changes in theory, but it is the accumulation and increase in these quantitative changes that lead to a change in grassland quality, which leads to grassland degradation [45]. Since the 1960s, the distribution area of *F.* *sibiricum* grassland has decreased, and the height, coverage and abundance of grass communities have also decreased significantly [46]. At present, the urgent problems to be solved in this grassland are poor forage quality and low yield. However, silage is an effective way to improve the utilization rate of *F.* *sibiricum* grass resources [47]. It is of great significance in theory and practice to provide a scientific basis for the rational use and improvement of *F.* *sibiricum* grasslands and other further research.

## Concluding remarks

In brief, *F.* *sibiricum* has been successfully used as a folk medicine for thousands of years. Research on the pharmacological and toxicological effects, medicinal chemical components of *F.* *sibiricum* is summarized and presented in this paper. Compounds in multiple phytochemical classes have been isolated from this medicinal plant. Available pharmacological studies on the compounds and crude extracts indicated broad biological effects of *F.* *sibiricum*, which provides a theoretical basis for the further research and development of *F.* *sibiricum* for food and medicine applications. First, in comparison to antibiotics (secondary metabolites produced by microorganisms or higher plants and animals) and artificial synthetic compounds, herbal medicines provide alternative therapy for human diseases with numerous unsurpassed characteristics, including fewer undesirable effects, lower costs, better compatibility and easier accessibility. Second, in today's green food development era, economic losses, such as chemical contamination of meat and eggs of animals and declining product quality, should cause animal husbandry production concerns. In addition, the development of a Chinese herbal medicine as fodder to prevent and treat the disease of livestock and poultry has practical implications that are profound for improving the environmental friendliness of livestock products and increasing the export of foreign exchange. Third, bacteriostatic experiments proved that the roots of *F.* *sibiricum* had little bacteriostatic effect. In addition, most chemical constituents are present in the flowers, with lower concentrations in leaves and the lowest concentrations in roots [48]. Hence, it is recommended that the medicinal portion of the herb be changed from whole plant to the aboveground part [49]. Fourth, because of the low solubility and poor bioavailability of *F.* *sibiricum*, flavonoids are not easily absorbed by the body [50]. Based on the good solubilization of polyethylene glycol, the herb it was made into drops, which improved its water solubility and bioavailability and increased its stability [51]. Furthermore, the optimum preparation technology of flavone dripping pills was selected by orthogonal design, which ensured drug quality and efficacy. Moreover, the suppository based on the water-soluble polyethylene glycol has the capacity of releasing a large amount of the herbal constituents, which is beneficial for rectal administration. Finally, acute and chronic toxicity should be comprehensively studied to establish safety and toxicological limits and provide guidance for clinical applications.

As a traditional Chinese medicinal material, *F.* *sibiricum* is of great significance to modernize Chinese medicine. The scientific theory and medicinal value of *F.* *sibiricum* were introduced, and the clinical pharmacology potential and significance of *F.* *sibiricum* were affirmed. At present, research has involved chemical components, pharmacological effects, clinical applications and other aspects. Simultaneously, drug development of the related bioactive compounds also laid the foundation for future clinical applications and the pharmaceutical market. However, some aspects still require scientific evaluation and exploration. There are current gaps in the scientific literature that need to be further investigated to accelerate ongoing scientific and clinical research. As a result, further research needs to be conducted. In summary, *F.* *sibiricum* has good development prospects and is of great significance to the progress of the medical and pharmaceutical industries.

## Data Availability

Information on *F.* *sibiricum* was gathered from various sources, including books on traditional Chinese herbal medicine and scientific databases such as PubMed, Google Scholar, Science Direct, Baidu Scholar, CNKI and other professional websites.

## Abbreviations

*F.**sibiricum*: *Filifolium**sibiricum**(L.*)*Kitam*
VRSA: Vancomycin-resistant *Staphylococcus* *aureus*
ESBLs: Extended-spectrum enzymes
AmpC: Cephalosporin enzyme
G^+^: Gram-positive
G^−^: Gram-negative
MRSA: Methicillin-resistant *Staphylococcus* *aureus*
MRCNS: Methicillin resistant coagulase negative Staphylococci
HPLC: High performance liquid chromatography
NMR: Nuclear magnetic resonance
GC–MS: Gas chromatography–mass spectrometry
MIC: Minimum inhibitory concentration
MBC: Minimum bactericidal concentration
NO: Nitric oxide
COX-2: Cyclooxygenase-2
iNOS: Inducible nitric oxide synthase
NAG: *N*-Acetyl-amino group glucosaminidase
UCr: Urinary creatinine
IL-2: Interleukin-2
ELISA: Enzyme-linked immunosorbent assay
CGRP: Calcitonin gene-related peptide
UHPLC–MS: Ultra-high-performance liquid chromatography–mass spectrometry
QC: Quality control
LLOQ: Lower limit of quantitation
LLOD: Lower limit of detection
R^2^: Regression parameters
S/N: Signal-to-noise ratio
GPT: Glutamic pyruvic transaminase
NFN: Non-protein nitrogen

## References

[1] National Compilation of Chinese Herbal medicine. National Compilation of Chinese Herbal medicines. Beijing: People’s Medical Publishing House, 1975.

[2] Singh A, Gautam PK, Verma A, Singh V, Shivapriya PM, Shivalkar S, Sahoo AK, Samanta SK (2020). Green synthesis of metallic nanoparticles as effective alternatives to treat antibiotics resistant bacterial infections: a review. Biotechnol Rep.

[3] Yan D, Xianyuan W, Hong Z, Juan Z (2008). Study on bacteriostatic effect of Chinese herbal medicine on MRSA clinical strains. Chin Nursing Res.

[4] Juntao Q, Changsheng R, Wenwei S (1996). Crude drug identification of *Filifoliumsibiricum*(L.)Kitam. J Chin Med Mater.

[5] Sun H, Song Y, Hou X, Zhang Y (2008). Study on micromorphological characteristics of Plant fruit in chrysanthemum. Lishizhen Med Mater Med Res.

[6] Xing F, Tingcheng Z (1992). A preliminary study on biomass and net primary productivity of *Filifoliumsibiricum*(L.) Kitam grassland in eastern Inner Mongolia. J Plant Ecol Geobot.

[7] Shaocheng W, Xianzhi L, Xiucheng G (1994). Resource features of Hulun Buir meadow grassland in China. J Tea Sci.

[8] Shipeng Y, Zongyuan Z (1982). The preliminary analysis of differentiation of *Filifoliumsibiricum*(L.) Kitam Steppe in mountainous region of the east and west sides of the Greater Khingan. Ser Phytoecol Geobot.

[9] Xuefeng S, Zhenwan Z, Yong S (1991). The compartment of vegetation of XiIin GoI Prairie in Inner Mongolia. Acta Bot Boreali-Occidentalia Sin..

[10] Hongmei L, Jie LI, Lili W, Jianning Z, Hui W, Dianlin Y (2018). The effect on Baikal needlepoint herb and Soil stoichiometric characteristics by nitrogen addition. Acta Pratacult Sin.

[11] Xing F, Jianhe L (1996). Dynamic Analysis of underground plant quantity and growth rate in *Filifoliumsibiricum*(L.)Kitam. grassland. Pratacult Sci.

[12] Shihuang C (1992). The distribution regularities and characteristics of vegetation underground biomass in Inner Mongolia Grassland. J Inner Mong Agric Animal Husbandry Coll.

[13] Taikang H, Zhizun D, Shouxun Z (2001). Compendium of modern Materia Medica.

[14] Peiyun F (1995). A key list of plants from the Northeast.

[15] Jingshi W, Li L (1980). Experimental study on microcapsule of *Filifoliumsibiricum* oil by complex coacervation. J Mudanjiang Med Univ.

[16] Antibacterial action and Clinical application of *Filifoliumsibiricum*(L.)Kitam. Chin Herbal Med Newsl. 1976,(06):33–49.

[17] Youchang Z, Decheng W, Jingfu L (1989). Medicinal plants of northeast China.

[18] Lingyan L, Lina A, Mingjie W, Zhenqi L (2002). Experimental study on the medicinal herb *Filifoliumsibiricum*(L.)Kitam. Heilongjiang Animal Sci Vet Med.

[19] Wang D (1984). Study on the effective components of *Tanacetum**sibiricum* L. J Pharm.

[20] Mingming L, Yuting S, Tao L, Zhuo J (2014). Study on chemical component of *Filifoliumsibiricum*(L.)Kitam (II). Heilongjiang Med J.

[21] Qiuhong W, Yujie L, Yang S, Shaowa L, Haixue K (2012). Study on the chemical composition of anti-infection effective parts of *Filifoliumsibiricum*(L.)Kitam(I). Chin Herbal Med.

[22] Zhenjie Z, Youmin W, Xiaowen Z, Xingsen L (1989). Study on chemical constituents of volatile oil from *Filifoliumsibiricum*(L.)Kitam. Acta Bot Boreali-West.

[23] Dong W, Mingyuan L, Haishan W (1986). Analysis of chemical components of volatile oil in *Tanacetum**sibiricum* L. by gas chromatography–mass spectrometry. Chinese Medicine Information.

[24] Junchan Q, Li C, Tao L, Yanyan J (1998). The determination of total flavonoids of *Filifoliumsibiricum*(L.)Kitam. Chin Trad Herbal Drugs.

[25] Haihong S, Dong W, Rui W (2013). Orthogonal experiment was conducted to optimize the extraction process of Scopoletin from *Filifoliumsibiricum*(L.)Kitam. Heilongjiang Sci Technol Inform.

[26] Mingming L, Zhenxing F, Yuanyuan Z, Dong W (2014). Study on chemical constituents of *Filifoliumsibiricum*(L.)Kitam. Heilongjiang Pharm.

[27] Yujie L, Qiuhong W, Xiaodong Y, Bingyou Y, Haixue K (2011). Study on the chemical composition of anti-infection effective parts of the *Filifoliumsibiricum*(L.)Kitam (II). J Chin Med.

[28] Lemmens KJA, van de Wier B, Koek GH, Köhler E, Drittij M-J, van der Vijgh WJF, Bast A, Haenen GRMM (2015). The flavonoid monoHER promotes the adaption to oxidative stress during the onset of NAFLD. Biochem Biophys Res Commun.

[29] Qiuhong W, Yujie L, Shaowa L, Bingyou Y, Haixue K (2011). Screening of active sites of resistance to drug resistant bacteria in *Filifoliumsibiricum*(L.)Kitam. J Chin Med Mater.

[30] Qiuhong W, Yujie LIU, Shaowa L, Xu M, Haixue K (2011). Study on the antibacterial action of *Filifoliumsibiricum*(L.)Kitam. Chin J Exp Trad Med Formula.

[31] Haixue K, Shaowa L, Yujie L, Qiuhong W (2011). The effect of pyelonephritis in rats by anti-infective part of *Filifoliumsibiricum*(L.)Kitam. Chin J Exp Trad Med Formula.

[32] Valenzuela B, Imarai M, Torres R, Modak B (2013). Immunomodulatory effects of the aromatic geranyl derivative filifolinone tested by the induction of cytokine expression. Dev Comp Immunol.

[33] Shaowa L, Yujie L, Yonghai M, Qiuhong W, Bingyou Y, Haixue K (2011). Study on analgesic and antipyretic effects of *Filifoliumsibiricum*(L.)Kitam on effective parts against infection. Acta Chin Med Pharmacol.

[34] Bi Q, Yaodong L, Laibing W, Ruiting M, Junchan Q (2019). Study on antipyretic, expectorant and antitussive effects of flavone dropping Pills of *Filifoliumsibiricum*(L.)Kitam. Guiding J Trad Chin Med Pharm.

[35] Yujie L, Qiu-hong W, Hong-bin X, Bo T, Shaowa L, Haixue K (2012). A preliminary study on the pharmacological effects of total flavonoids in *Filifoliumsibiricum*(L.)Kitam on treating chronic bronchitis. Chin Trad Patent Med.

[36] Shiying N, Denghua Z (2016). The related research and progress of *Filifoliumsibiricum*(L.)Kitam. J Inner Mongolia Med Univ.

[37] Xiuyu W (1979). A preliminary study on pharmacology of *Filifoliumsibiricum*(L.)Kitam. Heilongjiang Med J.

[38] Shaowa L, Dan X, Yuyan G, Qiu-hong W, Shuang S, Haixue K (2017). Pharmacokinetics of the three active components in plasma of rats after oral administration of the effective parts of *Filifoliumsibiricum*(L.)Kitam based on UPLC–MS. Chin J Exp Trad Med Formulae.

[39] Xiaoqi S, Junchan Q, Ruiting M, Laibing W, Bi Q (2020). Determination of eriodictyol and apigenin in *Filifoliumsibiricum*(L.)Kitam by variable wavelength HPLC. J Inner Mongolia Norm Univ (Chinese edition).

[40] Yuanjun Z, Dan S, Baizhu W, Zhongjie S, Xiaohui Y, Yanshu L. Floristic features and vegetation classification of the Hulun Buir Steppe in North China: geography and climate-driven steppe diversification. Global Ecol Conservation. 2019;20. 10.1016/j.gecco.2019.e00741.

[41] Shu-hua Z, Rui-lei C, Meng-li Z, Guo-dong H, Kun W (2008). Quantitative division and establishment of dynamic model of grassland degradation series of *Stipa**baikal* + *Filifoliumsibiricum*(L.)Kitam. Arid Land Res.

[42] Xinyi L. Grassland protection and utilization in Greater Khingan Mountains[A].Grassland Supervision Center of ministry of Agriculture, Chinese Society of Grasses. In: Proceedings of China Grassland Development Forum 2009[C].Grassland Supervision Center of ministry of Agriculture, Chinese Society of Grasses: Chinese Grassland Society, 2009:3.

[43] Tong C, Wu J, Yong S, Yang J, Yong W (2004). A landscape-scale assessment of steppe degradation in the Xilin River Basin, Inner Mongolia. China J Arid Environ.

[44] Biao Q, Degang Z, Lingling D, Li Z, Xi W, Yuhai Y (2005). Characteristics of community diversity of Degraded alpine arid grassland plant. J Gansu Agric Univ.

[45] Limin Y, Renzhong W, Jiandong L (1999). Effects of disturbance gradient grazing in main communities of Songnen Plain on plant diversity. Acta Agrestia Sin.

[46] Guixiang L, Zhongshan S, Xiaotian W. A study on the dynamics of *Filifoliumsibiricum*(L.)Kitam grassland over the past 40 years—a case study of Xilingol Grassland. In: 2006 proceedings of development Forum on China Grass Industry. Chin Grassland Soc 2006:365–369.

[47] Xing F, Hongzhang Z, Shaomin T, Zhancheng J (1995). Nutrition dynamics and rational utilization of *Filifoliumsibiricum*(L.)Kitam grassland in Keyouqianqi Line, Inner Mongolia. China Grassland.

[48] Shaowa L, Min Z, Shuang Z, Zhili C, Haixue K, Qiuhong W (2008). Study on HPLC Characteristic map of effective parts of *Filifoliumsibiricum*(L.)Kitam and determination of 5 components. Chinese Herbal Med.

[49] Jingshi W, Zhiguo Y, Sijun Q, Xiufen W (1979). A preliminary study on the pharmacology of *Filifoliumsibiricum*(L.)Kitam. Chin J Pharm.

[50] Juanjuan Z, Jun Y, Yan X. Improvement strategies for the oral bioavailability of poorly water-soluble flavonoids: An overview. Int J Pharm. 2019;570. 10.1016/j.ijpharm.2019.118642. 10.1016/j.ijpharm.2019.118642 31446024

[51] Xianhui W, Wenxue M, Junchan Q (2011). Study on preparation technology of flavone dropping pills of *Filifoliumsibiricum*(L.)Kitam. J Med Pharm Chin Minorities.

